# MDM2 as a therapeutic target in advanced biliary tract cancers

**DOI:** 10.1093/oncolo/oyaf094

**Published:** 2025-05-27

**Authors:** Kristen R Spencer, Gentry G King

**Affiliations:** Department of Medicine at NYU Grossman School of Medicine, NYU Langone Perlmutter Cancer Center, New York, NY 10016, United States; Fred Hutchinson Cancer Center, University of Washington, Seattle, WA 98109, United States

**Keywords:** biliary tract cancer, cholangiocarcinoma, gallbladder cancer, *MDM2* amplification, oncogene, MDM2-p53

## Abstract

Biliary tract cancers (BTCs) are a heterogeneous group of tumors arising from cells in the bile ducts and gallbladder. The 5-year overall survival rate for all BTC stages combined is ~20%, and treatment options for patients with unresectable disease are limited, leaving an unmet clinical need. In recent years, significant efforts have been made to refine and implement targeted therapeutic approaches for patients with BTC. The adoption of early and comprehensive molecular profiling is crucial to identifying patients who may be candidates for effective targeted therapies. Characterization of the molecular landscape of BTCs led to the identification of murine double minute 2 homolog gene (*MDM2)* amplification across all BTC subtypes. The MDM2 protein is a critical negative regulator of p53 stabilization and activity that is an emerging actionable biomarker in BTCs. There are multiple therapeutic approaches that aim to target MDM2 activity, thereby restoring the intrinsic tumor suppressor function of p53 and halting oncogenesis. However, these have been limited by our evolving understanding of the role of MDM2 in BTC pathogenesis. Here, we offer a review of the current understanding of the role of MDM2 in BTC biology and its therapeutic implications.

**Implications for Practice**

Biliary tract cancers (BTCs) are a heterogeneous group of tumors with poor prognosis and limited treatment options. However, BTCs have a high number of targetable alterations, including amplifications of the *MDM2* gene, which encodes murine double minute 2 homolog (MDM2) protein, a critical negative regulator of the tumor suppressor p53. The integration of comprehensive molecular profiling into the treatment pathway is imperative to ensure access to potential targeted therapies for patients with BTC.

## Introduction

**Figure 1.**
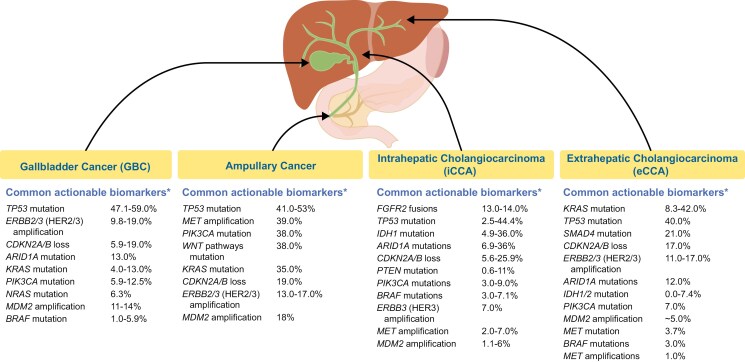
BTC subtypes and associated biomarkers.^1,8-17^ * Additional tumor-agnostic targetable alterations in BTCs include *RET* and *NTRK* gene fusions, MSI-H/dMMR, *TMB-H*, and *HER2* overexpression. Abbreviations: BTC, biliary tract cancer; CDKN2A/B, cyclin-dependent kinase inhibitor 2A/B; FGFR2, fibroblast growth factor 2; HER2/3, human epidermal growth factor 2/3; MDM2, murine double minute 2; MET, mesenchymal epithelial transition factor; MSI-H/dMMR, microsatellite instability–high/mismatch repair–deficient; PIK3CA, phosphatidylinositol-4,5-bisphosphate 3-kinase, catalytic subunit alpha; PTEN, phosphatase and tensin homolog; SMAD4, suppressor of mothers against decapentaplegic 4; TMB-H, tumor mutational burden–high; WNT, wingless-related integration site. Image adapted with permission from Lamarca A, et al. *ESMO Open*. 2022;7:100378.

Biliary tract cancers (BTCs) are a heterogeneous group of rare malignancies that can arise in the intrahepatic and extrahepatic bile ducts, the gallbladder, and the ampulla of Vater.^1^ The incidence of BTCs was <5 cases per 100 000 persons in the United States between 2013 and 2017, but it has been increasing in the past 2 decades.^2,3^ The most common subtype of BTC is cholangiocarcinoma (CCA; cancer of the bile ducts), which consists of intrahepatic CCA (iCCA; second-order bile ducts in the liver; 1.49 cases per 100 000 persons), and extrahepatic CCA (eCCA; 0.96 cases per 100 000 persons). eCCA can be further divided into perihilar CCA and distal CCA.^3-5^ Gallbladder cancer (GBC; cancer of the gallbladder or cystic duct) is the second-most frequent subtype of BTC, with an incidence of 1.11 cases per 100 000 persons.^3^ In contrast, ampullary cancer (cancer of the ampulla of Vater) is the rarest BTC subtype (0.45 cases per 100 000 persons) and is often excluded from studies and trials.^3,4,6,7^ In addition to differences in BTC anatomic location, there is also considerable molecular heterogeneity both within and between subtypes (Figure 1).^18^ This molecular heterogeneity may be influenced by both cell-autonomous (eg, genetic) and non–cell-autonomous elements (eg, microenvironment).^18,19^

There is a significant unmet clinical need for this growing patient population, as patients with BTC are faced with diagnostic challenges and have limited therapeutic options, both factors that contribute to the poor prognosis associated with BTCs.^4,20-22^ BTCs often present without any specific symptoms, and diagnosis often requires a combination of laboratory and imaging tests, which results in most cases being diagnosed at an advanced stage.^4,5,23,24^ This may contribute to the poor 5-year relative survival rates, which are cumulatively (irrespective of stage of diagnosis or anatomic site) 15.2% and are as low as 3.0% for those diagnosed with metastatic BTC.^25^ The addition of immunotherapies to standard first-line chemotherapy regimens has only marginally improved outcomes for patients with advanced disease: durvalumab in combination with chemotherapy (gemcitabine and cisplatin) was associated with a median overall survival (OS) of 12.9 months compared with 11.3 months for chemotherapy alone (hazard ratio [HR] 0.76, 95% confidence interval (CI), 0.64-0.91]),^26^ whereas pembrolizumab in combination with chemotherapy was associated with a median OS of 12.7 months compared with 10.9 months for chemotherapy alone.^27^ Therefore, the unmet need for more efficacious treatments for these patients remains high.

The use of integrative genomic approaches to further understand the pathogenesis of BTC tumors has identified multiple dysregulated oncogenic pathways and paved the way for targeted therapeutics that may provide better outcomes for patients with BTC than standard chemotherapy-based regimens alone.^5,28-30^ Targeted therapy options are now available for patients with advanced BTC (Table 1); therefore, molecular testing is recommended as part of the workup for unresectable or metastatic tumors.^31^ The alterations targeted by these therapies include high microsatellite instability, mismatch repair deficiency, high tumor mutational burden, fusions or rearrangements in *RET, FGFR2* or *NTRK,* overexpression or amplification of *HER2* (*ERBB2*), and mutations in *IDH1* and *BRAF.*^31^ Of great clinical interest, therapies targeting *HER2*, *KRAS*, *BRAF*, and *MDM2* are currently under investigation.^5,32^

**Table 1.** Guideline-recommended targeted therapies for unresectable or metastatic BTC.^a^^,16,17,31^

Target biomarker	Recommended therapies
***NTRK* gene fusion positive**	Larotrectinib Entrectinib Repotrectinib
**MSI-H/dMMR**	Pembrolizumab^b^ Dostarlimab-gxly^b,c^
**TMB-H** ^d^	Nivolumab + ipilimumab^b,e^ Pembrolizumab^b^
***BRAF* V600E mutation**	Dabrafenib + trametinib
***FGFR2* fusion or rearrangement** ^f^	Pemigatinib Futibatinib
***IDH1* alterations** ^f^	Ivosidenib
**HER2 positive**	Fam-trastuzumab deruxtecan-nxki (IHC3+) Trastuzumab + pertuzumab Tucatinib + trastuzumab Zanidatamab-hrii (IHC3+)
***RET* gene fusion positive**	Pralsetinib Selpercatinib
***KRAS* G12C mutation positive**	Adagrasib

^a^Recommendations depend on the line of therapy. ^b^For patients who have not been previously treated with a checkpoint inhibitor when used as subsequent-line therapy because there is a lack of data for use of immunotherapy in patients who have previously been treated with a checkpoint inhibitor. ^c^Dostarlimab-gxly is a recommended treatment option for patients with MSI-H/dMMR recurrent or advanced tumors that have progressed on or following prior treatment and who have no satisfactory alternative treatment options. ^d^Suggested cutoff score of ≥ 10 somatic mutations per megabase, based on an FDA-approved test per the approved indication for pembrolizumab. ^e^In the subsequent-line setting, this regimen is an option for patients with disease refractory to standard therapies or who have no standard treatment options available. ^f^For CCA only.

Abbreviations: BTC, biliary tract cancer; CCA, cholangiocarcinoma; dMMR, mismatch repair deficient; FDA, US Food and Drug Administration; IHC, immunohistochemistry; MSI-H, microsatellite instability–high; TMB-H, tumor mutational burden–high.

In this review, we discuss investigational targeted therapies for BTC, the challenges of molecular profiling to identify therapeutic targets in BTC, and the potential of therapeutic approaches targeting murine double minute 2 homolog (MDM2), an E3 ubiquitin ligase that inhibits p53, for BTC treatment.^8^

## Molecular characteristics of BTC

Comprehensive integrated genomic, epigenomic, and transcriptomic analyses have identified molecular alterations that correlate with differences in BTC tumor etiology, tumor biology, clinical features, and prognosis.^28-30^ Alterations in genes that affect cell growth and differentiation (eg, *HER2*), proliferation and survival, cell cycle regulation, DNA damage repair and genomic instability (eg, *TP53*, *MDM2*, *BRCA1/2*), epigenetic regulation (eg, *IDH1/2*, *BAP1*, *TET1-3*), kinase signaling (eg, *KRAS*, *ERBB1-3*, *PTEN*, *BRAF*, *PIK3CA*, *FGFR1-3*), and immune dysregulation (eg, *JAK-STAT3*), among others, have been identified in tumors from patients with BTC.^5,29,33^

The tumor-suppressing protein p53, encoded by the gene *TP53*, is one of the most frequently mutated genes across BTC subtypes,^28-30,34^ and alterations in *TP53* are associated with a poor prognosis. In a study by Nakamura et al that compared whole-exome sequencing in 239 BTC tissue samples with paired normal tissue, alterations in the *TP53* gene cluster (*ATM*, *MDM2*, *CDKN1A*, and *TP53*) were identified in 33.9% of patients with BTC.^29^

## Therapeutic implications for the MDM2-p53 axis in BTC

**Figure 2.**
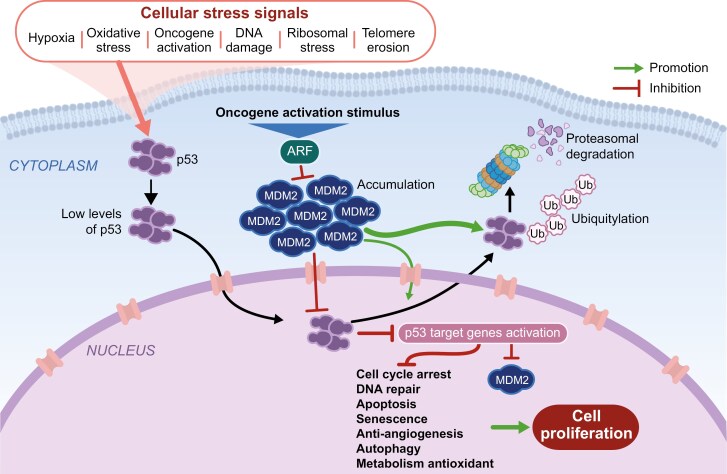
Overview of the MDM2-p53 pathway.^35-39^ Schematic of the MDM2-p53 pathway in a cancer cell. Under normal conditions, induction of cellular stress signals leads to p53 translocation to the nucleus to induce expression of target genes involved in cell cycle arrest, DNA repair, apoptosis, senescence, anti-angiogenesis, autophagy, and metabolism. MDM2 regulates the levels of intracellular p53 through ubiquitination and is itself negatively regulated by p14ARF, which is activated by oncogenic stimuli. Ubiquitinated p53 is degraded by the proteosome. In cancer cells, *MDM2* amplification leads to elevated levels of MDM2 protein, which can interact with and prevent p53 from inducing expression of its target genes under cellular stress conditions. This in turn can drive oncogenesis. Abbreviations: ARF, ADP ribosylation factor; DNA, deoxyribonucleic acid; MDM2, murine double minute 2; p53, tumor protein p53; Ub, ubiquitin.

The MDM2 protein is part of the p53 signaling pathway (Figure 2)^8,29,40^; amplification of *MDM2* is a frequent oncogenic driver and has been reported across BTC subtypes, albeit less frequently than *TP53*.^8,29,40^

*MDM2* encodes an E3 ubiquitin–protein ligase that localizes to the cell nucleus and is the main negative regulator of the p53 tumor suppressor protein, which plays a critical role in protection against cancer proliferation.^35^ In normal, unstressed cells, levels of p53 are kept low partly by MDM2-mediated ubiquitination. Under these conditions, ubiquitinated p53 is degraded by the proteasome, and the p53 response is maintained in check.^35,36,41,42^ A wide range of antiproliferative responses can be invoked by the MDM2-p53 pathway, including cell cycle arrest, apoptosis, and regulation of DNA repair mechanisms. Activation of these vital processes in healthy cells typically occurs in response to numerous cellular signals that may arise during malignant cell cycle progression or throughout DNA repair processes.^36,41,43^ In tumor cells with increased amplification of *MDM2*, p53 is overly inhibited, leading to excessive downstream silencing of p53 function and a failure to activate the tumor suppressor response. This contributes to oncogenic transformation and rapid cell proliferation (Figure 2).^36,41,42^

As a main regulator of p53 function, the MDM2-p53 interaction is an attractive target for therapeutic drug development.^8,35,44,45^ The MDM2-p53 pathway has been extensively characterized, and it is recognized as the most altered pathway in a wide variety of cancers, including BTC.^40,46^ The incidence of *MDM2* amplification in some forms of BTC is relatively high: 11%-14% in GBC and 18% in ampullary cancer. However, *MDM2* amplification is less common in both iCCA (1%-2%) and eCCA (~5%; Figure 1). *MDM2* amplification has also been negatively associated with OS: patients with iCCA *MDM2* amplifications have a shorter median OS (16 months; *n* = 13) than patients without (28 months; *n *= 200; *P = *.017).^8,29^

While the specific role of *MDM2* amplification in BTC tumorigenesis has not yet been established, *MDM2* alterations have been shown to be sufficient to abrogate p53 function, leading to malignant cell transformation in the biliary tract.^35,36,41,47^

## *MDM2* amplification in BTCs in the clinic

The most common type of *MDM2* alteration is amplification, defined as an increase in *MDM2* copy number.^47-49^*MDM2* amplification is emerging as a targetable biomarker in BTCs, but identification of patients with this amplification may be challenging in practice given the absence of a standardized gene copy number (GCN) cutoff value for *MDM2*.^40,48,50,51^ For example, Kato et al used a cutoff of GCN ≥8 to characterize the *MDM2* amplification profile of patients with diverse malignancies, whereas phase 1 and 2 studies of the MDM2 inhibitor milademetan in patients with advanced or metastatic solid tumors have used a cutoff of GCN ≥12 to define *MDM2* amplification.^40,48,51^ Further prospective clinical trials of MDM2 inhibitors in BTC may help define a GCN cutoff that is linked to clinical outcomes, which could resolve this issue and provide insight into how to identify suitable patients for therapies targeting *MDM2* amplification.

## *MDM2* amplification as a prognostic biomarker in BTCs

Preliminary data indicate that *MDM2* amplification is a negative prognostic biomarker in BTC. In a study characterizing iCCAs, Japanese and Korean patients with *MDM2* amplification had a significantly shorter OS than those without (median OS, 16 vs 28 months; *P* = .017).^8^ Another study that analyzed samples from patients with advanced CCA showed that when patients with *MDM2* amplifications (*n* = 7) were compared with patients without (*n *= 151), patients with *MDM2* amplifications had shorter time to progression on first-line chemotherapy (HR 3.342; 95% CI, 1.1513-7.382; *P* = .003).^52^ Similarly, in a study characterizing GBCs using 244 samples from 233 patients, patients with *MDM2* amplification had a significantly shorter OS than patients with wild-type *MDM2* (median survival, 20 vs 34 months; *P* = .05).^53^ Additional follow-up studies are needed to confirm the prognostic implications of *MDM2* amplification in BTC in the setting of currently available therapeutic regimens and determine whether there are differences in its prognostic value across different subtypes of BTC.

## *MDM2* and coalteration in BTCs

Coalterations or comutations may be associated with improved or dampened treatment responses in patients receiving therapy directed toward presumed oncogenic drivers.^9^ A large-scale computational analysis of a clinicogenomic dataset identified several comutation patterns that impacted outcomes of targeted therapies across cancer types.^29^*MDM2* amplification has been variably observed alongside other genetic alterations depending on BTC subtype. For instance, in iCCA, loss of *SMAD4* expression was more commonly observed in patients with *MDM2* amplification.^8^ There was also a weak correlation between *MDM2* amplification and *TP53* mutations, with *TP53* mutations found in only 23% of *MDM2*-amplified iCCAs.^8^ However, other studies have shown that *MDM2* amplification was rarely found with *TP53* loss-of-function mutations, and these 2 types of genetic alterations were shown to be mutually exclusive in studies in patients with different BTC subtypes and in patients with GBC.^29,54,55^*KRAS* mutations and *MDM2* amplification also appeared to be mutually exclusive, with none of the *MDM2*-amplified iCCA cases having *KRAS* mutations in 1 study.^8^ More data on coalterations and their implications for patients with *MDM2*-amplified BTC will be of critical importance in selecting patients most likely to derive benefit from MDM2-targeted therapies and for selection of future combination strategies.^9^

## MDM2 antagonists in BTCs

MDM2 antagonists have been under development for over 15 years, with many agents performing unsuccessfully in clinical trials, and newer therapies currently under investigation (Table 2).^56,57^ Nutlins are small molecules that effectively disrupt the MDM2-p53 interaction by binding to the MDM2 binding pocket, leading to reactivation of the p53 pathway in both in vitro and in vivo models.^58,59^ The first MDM2 inhibitor tested in clinical trials was RG7112, a molecule designed with an optimized nutlin structure.^60,61^ In 2 phase 1 trials (1 in patients with liposarcoma and 1 in patients with leukemia), RG7112 showed increased p53 expression and produced a modest clinical benefit, and most patients had either stable disease or progressive disease. However, serious adverse events (AEs) were reported in 71% and 40% of patients (from the 2 trials) and neutropenia reported in 30% and 27% of patients.^62,63^ In vitro data suggested that the hematologic toxicities observed with RG7112 treatment may have resulted from excessive drug-mediated disruption of MDM2-p53 interactions, leading to p53 induction.^64^ As a result, apoptosis of hematopoietic progenitor cells occurred at a higher-than-normal rate during early stages of megakaryocytopoiesis, and delayed megakaryocyte maturation during later stages of this process ultimately impacted platelet production.^65^

**Table 2.** Clinical trial overview for select MDM2 antagonists.

MDM2-targeting agent	Core structure	Trial	Phase	Indications evaluated	Treatment arms	Results available
**Idasanutlin** **(RG7388)**	Pyrrolidine	NCT04029688 Terminated in 2024	1/2	Relapsed/refractory acute leukemias, solid tumors (pediatric and young adult patients)	Idasanutlin + chemotherapy Idasanutlin + venetoclax	Yes
**SAR405838** **(MI-77301)**	Spirooxindole	NCT01636479 Completed in 2018	1	Solid tumors with *TP53* mutation prevalence < 40% or WT • No patients with BTC	SAR405838 in different doses	Yes
		NCT01985191 Completed in 2016	1	Solid tumors with WT *TP53* and *RAS/RAF* mutations (≥ 1 with intrahepatic bile duct cancer)	SAR405838 + pimasertib (MEK1/2 inhibitor)	Yes
**Milademetan** **(DS-3032)**	Spirooxindole	NCT01877382 Completed in 2020	1	Advanced solid tumors, preferentially tumors associated with high prevalence of *MDM2* amplification (53/87 DDLPS)	Dose escalation of milademetan (n = 87) Dose expansion of milademetan (n = 20)	Yes
		NCT05012397 (MANTRA-2) Terminated in 2023	2	Advanced solid tumors with WT *TP53* and *MDM2* copy number ≥ 8	Milademetan	Yes
		NCT04979442 (MANTRA) Terminated in 2023	3	Advanced DDLPS	Milademetan (n = 86) • Trabectedin (n = 89, comparison arm)	Yes
**Brigimadlin**	Spirooxindole	NCT03449381 Phase 1a completed Phase 1b estimated completion in 2026	1	Advanced solid tumors • In a preliminary analysis, 28/54 (52%) patients had *MDM2* amplification and 10/54 (19%) had BTC	Brigimadlin in different doses	Yes
		NCT03964233 Phase 1a completed Phase 1b estimated completion in 2026	1	Advanced solid tumors • Preliminary analysis has 6 patients with BTC	Brigimadlin + ezabenlimab	Yes
		NCT05512377 Brightline-2 Estimated completion in 2026	2a/2b	Advanced cancer in biliary tract, pancreas, lung, or bladder with WT *TP53* and *MDM2* copy number ≥ 8	Brigimadlin	No
**Navtemadlin**	Piperidinone	NCT03787602	1b/2	Merkel cell carcinoma with WT *TP53*	Navtemadlin in different doses Navtemadlin in different doses + avelumab	Yes

Abbreviations: BTC, biliary tract cancer; DDLPS, dedifferentiated liposarcoma; MDM2, mouse double minute 2 homolog; MEK, mitogen-activated protein kinase; TP53, tumor protein p53; WT, wild-type.

SAR405838 is a novel small-molecule derivative of the MDM2 inhibitor MI-219, but in an optimized spirooxindole compound.^66^ When compared with nutlin-3a and MI-219 in a preclinical study, SAR405838 increased p53 activity and induced cell cycle arrest at much lower (nanomolar) concentrations, indicating greater potency and specificity compared with the earlier MDM2 antagonists.^67^ In a phase 1 clinical trial assessing SAR405838 in patients with solid tumors (including liposarcoma, gastrointestinal tumors, melanoma, non–small cell lung cancer and others), SAR405838 was clinically active, with stable disease in 56% of patients and progression-free survival in 32% at 3 months.^68^ While SAR405838 was better tolerated than first-generation nutlins, hematologic toxicities remained the primary dose-limiting factor, again attributed to the disruption of MDM2-p53 interactions.^68,69^

In recent trials of MDM2 inhibitors, intermittent treatment schedules have helped mitigate dose-limiting hematologic toxicities while maintaining efficacy.^51^ In a phase I trial (NCT01877382), the MDM2 antagonist milademetan (DS-3032), which inhibits the MDM2-p53 interaction at nanomolar concentrations in vitro, demonstrated antitumor activity in patients with advanced liposarcoma, solid tumors, or lymphomas when administered with an intermittent dosing schedule (260 mg once daily on days 1-3 and days 15-17 every 28 days). The rates of grade ≥ 3 AEs in patients receiving intermittent dosing were 15.0% for thrombocytopenia, 5.0% for neutropenia, and 0% for anemia, versus 36.2%, 20.3%, and 21.7%, respectively, in patients that received continuous dosing.^51^ While intermittent dosing may have helped reduce the occurrence of hematologic toxicities, baseline hematologic abnormalities may ultimately be a contraindication to therapy with MDM2 antagonists. In the MANTRA-2 phase 2 trial (NCT05012397) of milademetan in advanced or metastatic solid tumors, 1 of the 10 patients evaluable for efficacy had BTC. Interim results from this trial showed that this patient had a tumor that had a 29% reduction in diameter (a near-partial response) and was still on treatment after more than 32 weeks, as of the October 2022 cutoff.^70^

Brigimadlin (BI 907828) is an MDM2-p53 antagonist that directly interferes with the MDM2-p53 interaction, leading to p53 stabilization and restoration of wild-type p53 tumor suppressor function. Preclinical studies demonstrated durable tumor regression in a liposarcoma patient–derived xenograft mouse model.^71,72^ Brigimadlin was evaluated in 2 phase 1a/b dose-escalation/expansion trials as a monotherapy (NCT03449381) and in combination with the anti–programmed death-1 (PD-1) antibody ezabenlimab (NCT03964233) in patients with solid tumors, including advanced BTCs. In an analysis of data for patients with BTCs from these two trials, 6 patients (50%) in the monotherapy trial and 3 (43%) in the combination trial achieved stable disease. Of the 4 patients with BTC in the combination trial who responded, 1 notable response occurred in a patient with *MDM2*-amplified GBC with a partial response, with a maximum tumor shrinkage of 50% observed.^73^ The most common any-grade treatment-related AEs were nausea (68% for monotherapy, 70% for combination therapy), fatigue (54% for monotherapy, 39% for combination therapy), and neutropenia (52% for monotherapy, 34% for combination therapy). The most common grade ≥ 3 treatment-related AEs were neutropenia (25% for monotherapy, 24% for combination therapy) and thrombocytopenia (23% for monotherapy, 22% for combination therapy).^73^ These phase 1 findings were the basis for the Brightline-2 phase 2a/b study (NCT05512377) of brigimadlin in patients with advanced, *MDM2*-amplified, *TP53* wild-type BTC, pancreatic ductal adenocarcinoma, and other select solid tumors.^49,74^ This study has closed, and the brigimadlin development program across various oncology indications has been discontinued.

Navtemadlin (KRT-232) is another MDM2 inhibitor that is being investigated with or without anti–PD-1/anti–programmed death-ligand 1 (PD-L1) therapy in a phase 1b/2 trial (NCT03787602) in adult patients with Merkel cell carcinoma. It is a potent and selective orally available MDM2 inhibitor that aims to restore p53 activity and induce apoptosis of *TP53* wild-type tumors.^75^ Preliminary data from the phase 1b/2 trial showed a 25% confirmed overall response rate (ORR), a 38% unconfirmed plus confirmed ORR, and a 63% disease control rate in patients treated with 180-mg navtemadlin for a 5-days-on/23-days-off regimen in evaluable patients. Most treatment-emergent AEs were hematologic, but doses ≤ 180 mg were well-tolerated, with fewer dose reductions and longer treatment durations. Recruitment for this trial is ongoing.^75^

## Combination therapies targeting the MDM2-p53 pathway and other pathways of interest in BTCs

While precision oncology is an exciting option for many patients with advanced cancers, studies such as the NCI-MATCH trial have shown that few genes proposed as oncogenic drivers are successfully targeted by single-agent therapies, with responses limited by concurrent or emerging resistance alterations.^76^ As such, there are ongoing efforts to develop rational combination strategies to address mechanisms of resistance. Molecular testing is a critical component of this process, enabling physicians to better identify candidates for combination approaches.^76^

Combining MDM2 inhibitors with immunotherapies has shown some evidence of synergistic therapeutic effects.^77-79^ In the preliminary analysis of the brigimadlin phase 1a/b studies in patients with solid tumors, including BTCs (NCT03964233), partial response was noted in 4 of 6 patients who received the combination of brigimadlin and the anti–PD-1 antibody ezabenlimab, and in 3 of 10 patients who received brigimadlin monotherapy.^73^ Further data characterizing the predictors of response and resistance in patients with *MDM2*-amplified BTC enrolled in these and other clinical studies will provide the rationale for future combination strategies. The aforementioned phase 1b/2 trial investigating the effects of navtemadlin in Merkel cell carcinoma will also examine the effect of combining navtemadlin with avelumab in patients who were anti–PD-1/anti–PD-L1–naïve. In addition, a phase 1/2 clinical trial evaluated the activity of idasanutlin (a second-generation nutlin)^80^ in combination with either chemotherapy or the BCL-2 inhibitor venetoclax^81^ for the treatment of relapsed or refractory acute leukemias or solid tumors in pediatric or young adult patients; however, that trial was terminated due to insufficient tolerability and efficacy.^82^

## Conclusions

Patients with BTCs have a poor prognosis and face treatment challenges due to late diagnosis, high relapse rates, and limited effective therapeutic options. Despite the high number of targetable mutations found in these cancers, further therapeutic progress for this heterogeneous group of tumors is urgently needed. Preclinical and initial clinical data suggest that *MDM2* amplification could be a new target for treatment of BTC either alone or in combination with other available therapies. However, the role of MDM2 in BTC and mechanisms of response and resistance remain to be clarified.

## Data Availability

No new data were generated or analyzed in support of this review article.
